# The *Star Wars* Scroll Illusion

**DOI:** 10.1177/2041669515604060

**Published:** 2015-09-30

**Authors:** Arthur G. Shapiro

**Affiliations:** Department of Psychology, Collaborative for Applied Perceptual Research & Innovation (CAPRI), American University, Washington, D.C., USA

**Keywords:** *Star Wars*, perspective, Leaning Tower Illusion, 3D perception, illusion, Alderaan

## Abstract

The *Star Wars* Scroll Illusion is a dynamic version of the Leaning Tower Illusion. When two copies of a *Star-Wars*-like scrolling text are placed side by side (with separate vanishing points), the two scrolls appear to head in different directions even though they are physically parallel in the picture plane. Variations of the illusion are shown with one vanishing point, as well as from an inverted perspective where the scrolls appear to originate in the distance. The demos highlight the conflict between the physical lines in the picture plane and perspective interpretation: With two perspective points, the scrolling texts are parallel to each other in the picture plane but not in perspective interpretation; with one perspective point, the texts are not parallel to each other in the picture plane but are parallel to each other in perspective interpretation. The size of the effect is linearly related to the angle of rotation of the scrolls into the third dimension; the Scroll Illusion is stronger than the Leaning Tower Illusion for rotation angles between 35° and 90°. There is no effect of motion per se on the strength of the illusion.

The Leaning Tower Illusion (Kingdom, Yoonessi, & Gheorghiu, 2007a, 2007b, in press) won first place at the 2007 Best Illusion of the Year Contest and has been reproduced hundreds of times in news articles, blog posts, and other internet and print locations. The illusion consists of two identical static images of an object that tilts away from the viewer; when the images are placed side by side, one of the objects appears to tilt at a greater angle than the other. Kingdom et al. explained the phenomenon as a perspective illusion: That is, the two receding objects *fall* toward two different vanishing points. One implication of this principle is that any object that recedes into the distance should be able to create an effect similar to the Leaning Tower Illusion; the leaning tower effect opens a valuable method for studying how we construct a three-dimensional perceptual world from objects in a picture plane.

Here, I reproduce the leaning tower effect with scrolling text similar to the scrolling introduction in the *Star Wars* movies. The *Star Wars* scroll is perhaps one of the most iconic demonstrations of a simple object appearing to move into the distance, and therefore serves to illustrate the generality of the principle underlying the Leaning Tower Illusion. The basic version of the *Star Wars* Scroll Illusion is shown in Figure 1(a) (Movie 1, Part 1). Two identical scrolling texts are set side by side (or, if you will, the original scrolling text on the left and a clone on the right). In the picture plane, the texts scroll parallel to each other yet appear to diverge as they scroll into the distance: If you are sitting directly in front of the screen, the scroll on the left appears to veer to the left, while the scroll on the right appears to veer to the right. Figure 1(b) shows the vanishing points for each scrolling image and the perspective lines that radiate from the vanishing points. Figure 1(c) shows the triangles formed from the vanishing points and perspective lines without the scrolling text. The two triangles represent the lines in the picture plane and are clearly corresponding triangles shifted horizontally from each other.

**Figure 1.**
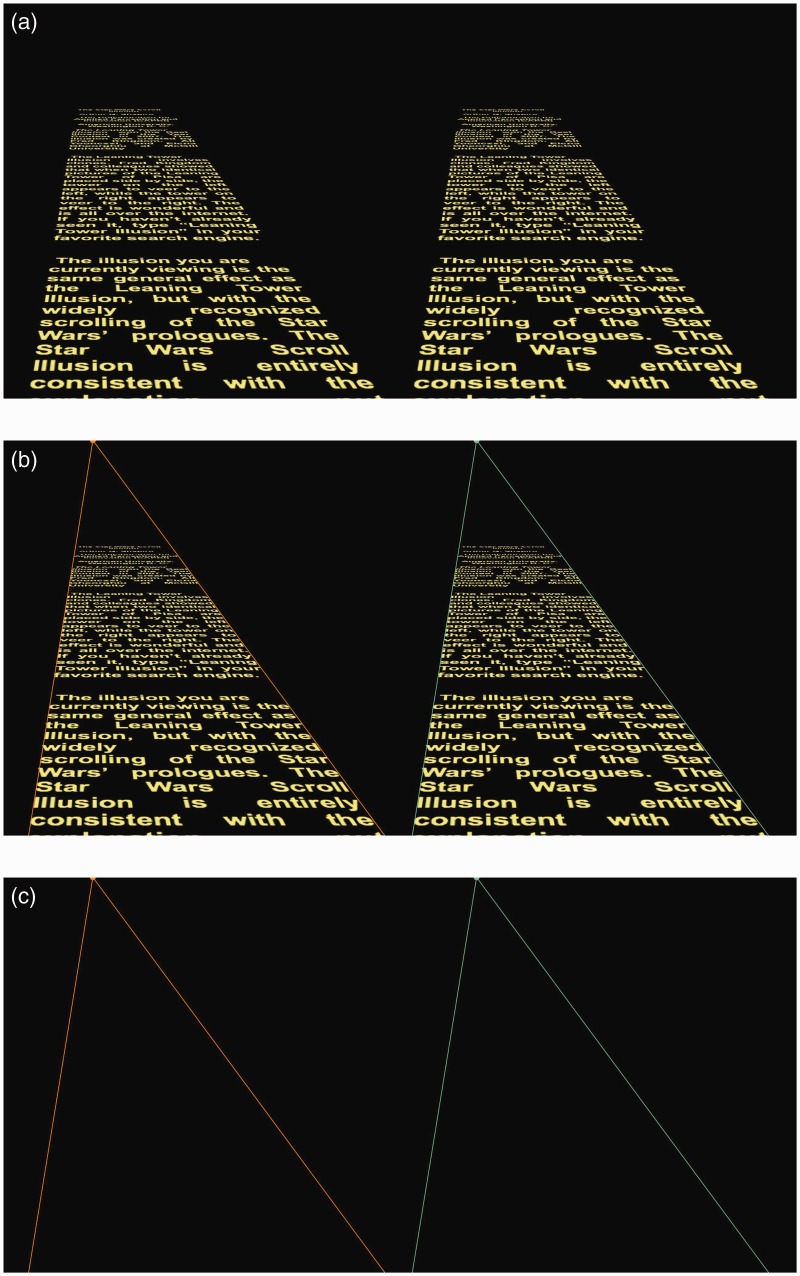
(see Movie 1, Part 1). Three still images from the *Star Wars* Scroll Illusion movie. (a) In the movie, the two identical scrolls begin at the bottom of the screen and appear to fade into the distance and diverge toward their separate vanishing points as they move toward the top of the screen. (b) Same as (a), but with vanishing points and constructor lines. (c) The vanishing points and constructor lines with text removed. The effect is more compelling in the movie than in the still images.

If the texts scroll to a single vanishing point, the reverse effect is created: The scrolling texts are parallel in perspective view but not parallel in the picture plane. This can be seen in Figure 2(a) to (c), which correspond to Figure 1(a) to (c), respectively. When shown in perspective view, the two scrolls seem to recede parallel to each other toward the vanishing point (Figure 2(a)), but the triangles formed from the vanishing points and perspective lines are not parallel to each other (see Figure 2(b) and (c)).

**Figure 2.**
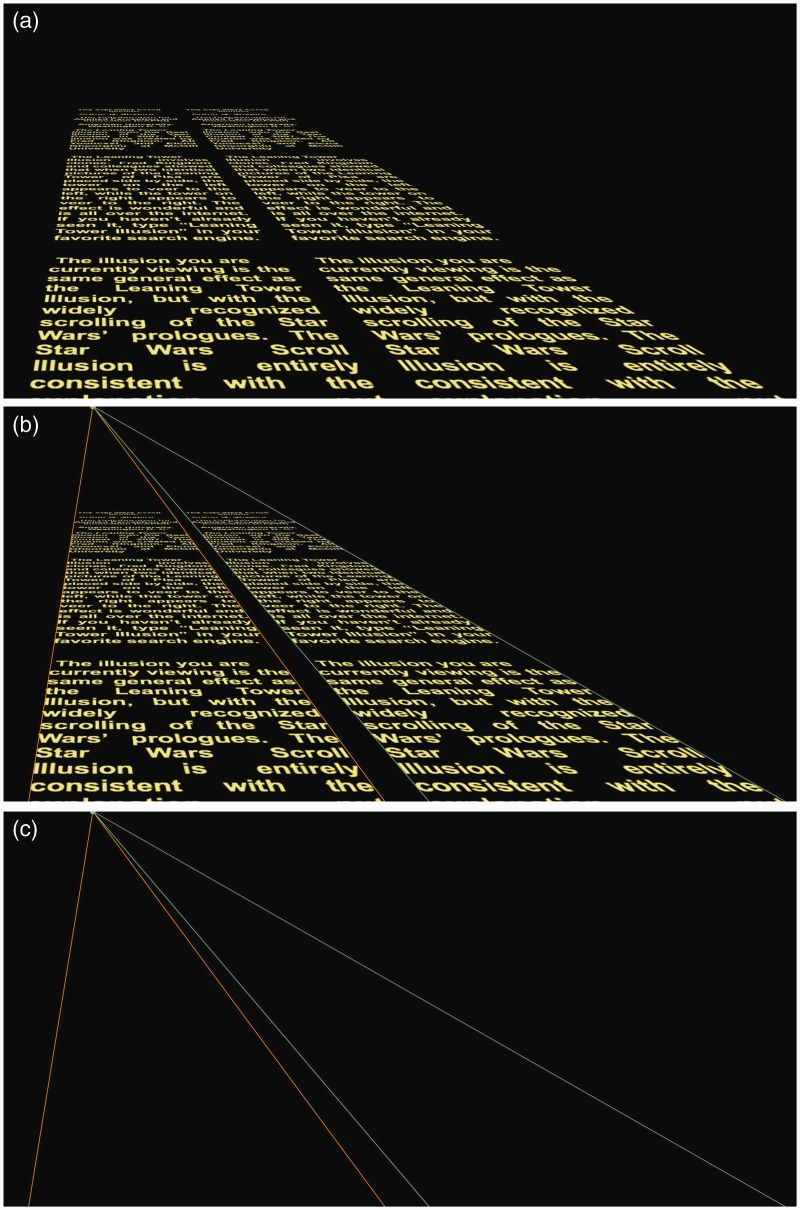
(see Movie 1, Part 2). The same as Figure 1 except that the two scrolls move toward a single vanishing point and therefore do not diverge as they appear to move into the distance.

The *Star Wars* Scroll Illusion functions from a variety of viewing angles. Figure 3 (Movie 2) shows the image as an *appearing* text: That is, the two scrolls appear to start scrolling in the distance, from a point at the bottom of the screen. The scrolls expand as they approach the top of the screen, as if the scrolling text were viewed from an imaginary planet inside a *Star Wars* movie (e.g., the view from Alderaan). When there are two *appearing* points, the scrolls appear to converge even though the lines are parallel in the picture plane; when there is a single appearing point, the texts appear to scroll in parallel. As with Figures 1 and 2, the effect appears much stronger in the movie than in the still figures.

**Figure 3.**
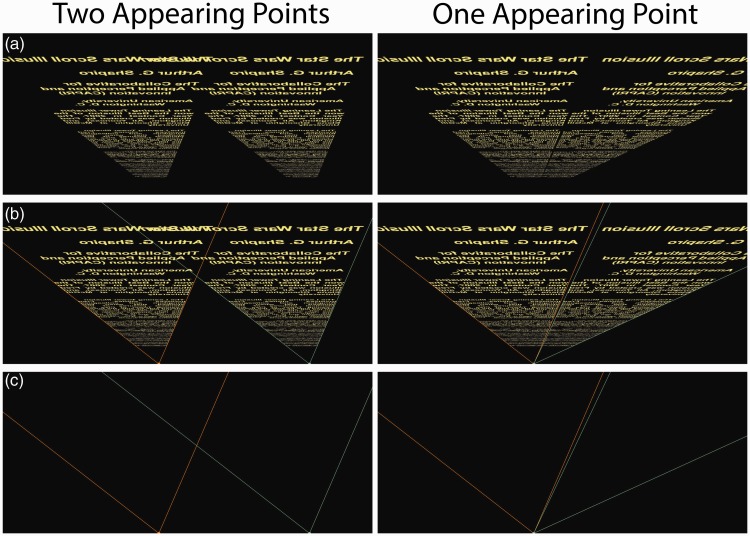
(see Movie 2). The view from Alderaan. The texts scroll upward from two appearing points (left column) or a single appearing point (right column). (a) With two appearing points, the texts appear to converge as they pass over the head of the observer. With one appearing point, the texts appear to run parallel to each other. (b) The scrolling texts from (a) with appearing points and constructor lines. (c) The appearing points and constructor lines without the scrolling texts.

## The Magnitude of the Effect Is Linearly Related to the Rotation Angle but Is Not Affected by Internal Motion

**Figure 4.**
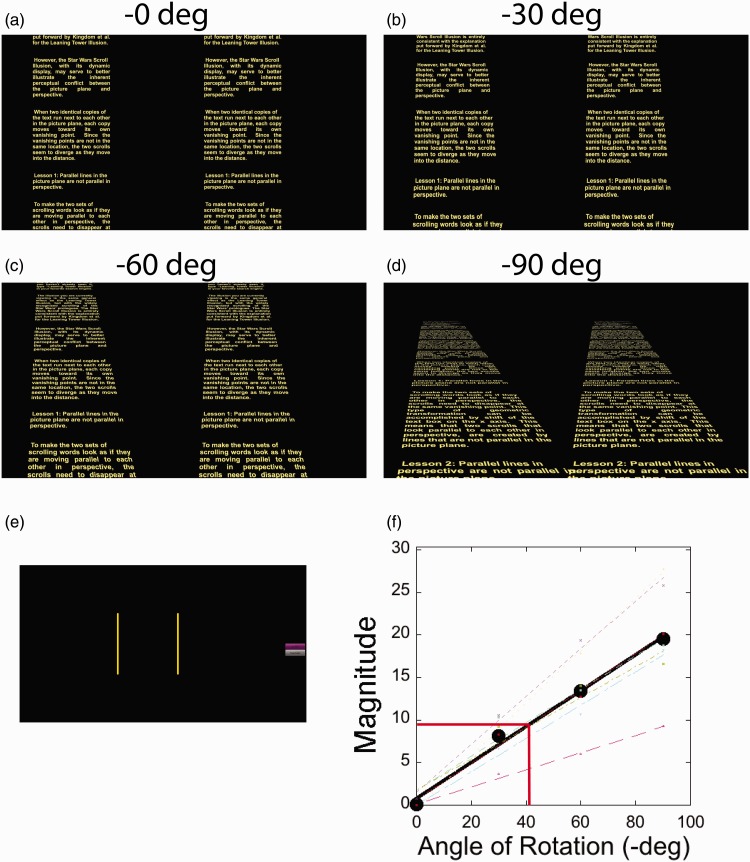
(see Movie 3). The effects of rotating the text in depth along the *x*-axis. (a) to (d) Still images of the scrolling text at 0°, −30°, −60°, and −90°. (e) Observers were asked to set the angle of yellow lines to match the directions that they perceived the text scrolls to move. (f) Magnitude of the effect (angle of right line − angle of left line) as a function of the angle of rotation along the *x*-axis. The filled circles indicate the average of all seven observers; the solid line is the best fit to the average. The individual symbols and dashed lines indicate the results from the seven observers. The red line indicates the average magnitude of the settings (and the corresponding angle of rotation) for the classic Leaning Tower Illusion.

As shown in Movie 3, the strength of the illusion is determined primarily by the rotation of the scrolling texts into the third dimension. Figure 4 shows the text rotated in perspective along the *x*-axis at 0, −30°, −60°, and −90° (the axis of rotation is not at the base of the scrolling text; hence, the rotation is similar to a slightly elevated camera angle, and 90° is not exactly perpendicular to the observer). The scrolling texts are identical but translated laterally from each other. At 0°, the texts appear to scroll in parallel in the picture plane. With each successive rotation, the apparent direction of the left scroll deviates further from the right scroll.

To measure the extent of the tilt as a function of *x*-axis rotation, we presented observers with five stimulus conditions: The four conditions in Figure 4 plus the classic image of the Leaning Tower Illusion from the Best Illusion of the Year Contest webpage. The seven participants in the study viewed the stimuli on a Sony OLED monitor at a distance of 50 cm. The stimuli were presented as 4-s clips. After each presentation, the clip was removed, and two lines appeared near the center of the screen on a black background (see Figure 4(e)). The observers pressed the portable keyboard to adjust the angles of two lines so that each line matched the observer’s sense of the apparent direction of one of the objects (i.e., the scrolling texts or the leaning towers). The observer could click a repeat button to see the 4-s clip; if the repeat button was pressed, the lines would disappear, and the clip would be presented again, followed by reappearance of the lines to be adjusted by the observer. When the observer was satisfied with the setting for the lines, she or he pressed an *accept setting* button, and the next trial commenced. The five stimulus conditions were presented six times each, for a total of 30 trials in the session. The magnitude of the effect was taken as the average of the angle set for the right line minus the average of the angle set for the left line; a value of zero indicates that the observer saw the tilt of both objects to be moving/leaning in the identical direction.

The results, presented in Figure 4(f), show that the magnitude of the effect changed linearly with the rotation into the third dimension (on average, Magnitude = 0.21 × Rotation_Angle + 0.79; *R*^2 ^= 0.99). All seven observers showed similar linear relationships (the lines fit to the individual data had an average *R*^2^ of 0.98); however, observers differed in the slopes of the best-fit line (from 0.1 to 0.31). Thus, while there was always a (remarkably) linear relationship between the perceived difference in tilt and the rotation angle, observers differed substantially in the reported magnitude of the effect. At this point, we cannot determine whether individual differences are due to observers’ perception or to a response bias in how observers set the orientation of the lines.

It seems likely, therefore, that the reason the *Star Wars* Scroll Illusion appears to have a larger magnitude than the Leaning Tower Illusion is that the images used to create the effects differ in their rotation into the third dimension. Observer settings for the Leaning Tower Illusion showed an average difference between the towers of 8.24°. The red line on the plot shows that the average difference produced by the *Star Wars* Scroll Illusion would be equivalent to a backward tilt of 35.2°—a magnitude equivalent to a relatively small rotation for the *Star Wars* Scroll Illusion (closer to Figure 4(b) than Figure 4(c)). The result suggests that the classic version of the Leaning Tower Illusion could be made stronger by shooting the image closer to the tower and therefore having a greater angle between the observer and the object. Another factor is that the images in the Leaning Tower Illusion seem to have a slight rotation along the *y*-axis, whereas the *Star Wars* Scroll Illusions shown here have no *y*-rotation. In the experiment, observers adjusted the left tower with a slight positive tilt, whereas for the *Star Wars* scrolls, observers adjusted the left scroll with a negative tilt.

The motion of the text itself as the scroll moves does not seem to be a contributing factor to the strength of the illusion. An experiment similar to that described earlier was conducted for eight naïve observers. There were four stimulus conditions: The text was either fully scrolled (long text) or partially scrolled (as if the text were only 1/3 of the display); the text was either still or moving. The stimulus conditions were presented eight times each, in random order, making 32 trials total. Figures all had an *x*-rotation of −90°. The average observer settings (averages ± standard error) for each condition are as follows: still, short text = 20.2 ± 4.9; moving, short text = 24.1 ± 4.1; still, long text = 23.5 ± 4.2; and moving, long text 22.7 ± 4.1. An analysis of variance with correlated samples showed no significant difference between the conditions (*F* = 2.17, *p* = .12). There was a trend for the still, short text condition to have less magnitude than the other conditions (*p* < .05 for five of the observers in individual data), but there could be a number of reasons for the short, still text to have a less defined angle. Also, as with the previous experiment, there was a strong correlation for observer settings for each condition; the correlation matrix had *r* values ranging .86 to .99. Thus, while the average values were similar across groups, individual observers either had a strong response bias or they were quite consistent in the magnitude of the effect relative to other observers.

## Conclusion

Many illusions *work* because they create a conflict between multiple reasonable interpretations. The central conflict in the *Star Wars* Scroll Illusion and the Leaning Tower Illusion seems to be between the picture plane and the perspective interpretations. With two perspective points, the scrolling texts are parallel to each other in the picture plane but not in perspective interpretation; with one perspective point, the texts are not parallel to each other in the picture plane but are parallel to each other in perspective interpretation. A major question for understanding how we see and interpret images concerns how the visual system can simultaneously maintain both of these types of representations.

Leaning Tower-type effects seem to give insight into how we construct perspective interpretations. Geometric illusions have a history of generating multiple types of explanation (Boring, 1942), so, undoubtedly, other explanations of this effect will continue to be offered. It is worth emphasizing here that explanations based on orientation contrast or internal cues cannot account for many of the central aspects of the illusion. Here, the motion of the scrolling text itself was shown not to have an effect on the magnitude of the display. In addition, the distortion occurs for the scrolling text (Panels 1A and 2A) but not for the perspective lines without the text (Panels 1C and 2C). Explanations of this type of geometric illusion in terms of local distortions of angles or other local cues would therefore require some way of creating the distortion in one condition but not the other. Yoonessi, Gheorghiu, and Kingdom (2008) present other reasons why non-perspective types of geometric explanations have a difficult time accounting for this type of effect. Lastly, many authors have argued that classes of geometric illusions result from misapplied depth cues (see Boring, 1942; Gregory, 2009). However, in the Scroll and Tower illusions, the depth information is not misapplied, as scrolls converging to two different vanishing points should actually be diverging from each other. These illusions can be explained not by the misapplication of perspective but rather by the conflict between two reasonable interpretations of the information in the image (perspective and picture plane).

Perhaps the most remarkable insight from the *Star Wars* Scroll Illusion and the Leaning Tower Illusion concerns the interaction between global construction of perceptual space and local perspective information. As commented on by Kingdom et al. (2007a, 2007b, in press), the differences between the perceived directions imply that the brain compensates for perspective across the whole image rather than for individual perspective cues. If this is so, then the Leaning Tower Illusion is part of a more general class of illusion in which local information deceives global operations, much like the Fraser Spiral (1908) and its successors have done with local direction cues and global shape processing. With the popularization of virtual reality devices such as the Oculus Rift, Kingdom et al.’s hypothesis may soon lead to an exciting new variety of phenomena that play on the interrelation between deceptive local three-dimensional cues and whole-image perspective.

## Supplementary Material

Supplementary material

## Supplementary Material

Supplementary material

## Supplementary Material

Supplementary material
